# Infantile-Onset Glutaric Acidemia Type I with Mild Hepatopathy: Clinical, Biochemical, and Molecular Characterization of an Iranian Pediatric Cohort

**DOI:** 10.3390/genes17040481

**Published:** 2026-04-18

**Authors:** Zahra Beyzaei, Bita Geramizadeh, Seyed Mohsen Dehghani, Sorour Inaloo, Ralf Weiskirchen

**Affiliations:** 1 Transplant Research Center, Shiraz University of Medical Sciences, Shiraz 7193711351, Iran; geramib@gmail.com; 2 Gastroenterology and Hepatology Research Center, Shiraz University of Medical Sciences, Shiraz 7193711351, Iran; dehghanism@sums.ac.ir; 3 Neuroscience Research Centers, Shiraz University of Medical Sciences, Shiraz 7193635899, Iran; sinaloo@sums.ac.ir; 4 Institute of Molecular Pathobiochemistry, Experimental Gene Therapy and Clinical Chemistry (IFMPEGKC), RWTH University Hospital Aachen, D-52074 Aachen, Germany

**Keywords:** glutaric acidemia type I, GCDH, mild hepatopathy, organic acidemia, genotype–phenotype correlation, pediatric metabolic disease

## Abstract

**Background:** Glutaric acidemia type 1 (GA1) is an autosomal recessive neurometabolic disorder caused by pathogenic variants in glutaryl-CoA dehydrogenase (GCDH), with variable clinical severity despite early biochemical detectability. Population-specific mutational spectra and genotype–phenotype correlations remain insufficiently defined in infantile-onset disease. Therefore, this study aimed to define the GCDH variant spectrum in GA1 patients with mild hepatopathy and assess genotype–phenotype correlations. **Methods:** We performed integrated clinical, biochemical, and molecular characterization of 15 unrelated patients with infantile-onset GA1. Whole-exome sequencing (WES) was performed for all participants, and the resulting data were compared with the reference sequence of the GCDH gene. **Results:** All patients presented within the first 6 months of life with macrocephaly, seizures, dystonia, and feeding difficulties. Neurological impairment and mild hepatopathy were variably observed, and one patient developed an acute encephalopathic crisis. Six homozygous GCDH variants were identified, predominantly missense. A common variant, c.541G>C (p.Glu181Gln), accounted for 73.3% of cases and defined a consistent phenotype of early macrocephaly and movement disorder with frequent mild hepatic involvement, suggesting regional enrichment and raising the possibility of a founder effect that warrants confirmation in future haplotype studies. A truncating variant, c.382C>T (p.Arg128Ter), was associated with severe early encephalopathy. Exon 6 represented a mutational hotspot. Biochemically, all patients showed elevated urinary glutaric and 3-hydroxyglutaric acids, increased glutarylcarnitine, and low-to-normal free carnitine, with higher metabolite levels in clinically more severe cases. All variants were pathogenic or likely pathogenic and extremely rare in population databases. **Conclusions:** This cohort reveals a striking predominance of the GCDH c.541G>C variant and establishes a clear biochemical signature with genotype-associated clinical patterns in infantile-onset GA1. These findings support a population-specific mutational spectrum, refine genotype–phenotype correlations, and underscore the importance of early molecular diagnosis to guide targeted neurological and hepatic monitoring as well as regional screening strategies.

## 1. Introduction

Glutaric acidemia type 1 (GA1; OMIM:608801) is a rare autosomal recessive organic acidemia resulting from a deficiency of glutaryl-CoA dehydrogenase (GCDH; EC 1.3.8.6), a mitochondrial enzyme involved in the degradation of L-lysine, L-hydroxylysine, and L-tryptophan [1]. Impaired activity of GCDH leads to the accumulation of characteristic metabolites, including glutaric acid, 3-hydroxyglutaric acid (3-OH-GA), glutaconic acid, and glutarylcarnitine (C5DC). Among these, glutaric acid and 3-OH-GA are considered neurotoxic and are implicated in the pathogenesis of the neurological phenotype [2,3].

**Figure 1:**
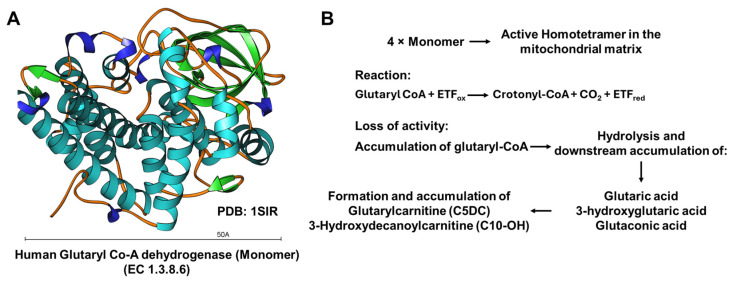
Overview of human glutaryl-CoA dehydrogenase (GCDH) function and metabolic consequences of loss of activity. (**A**) Structural representation of a human GCDH monomer based on the deposited three-dimensional structure (PDB: 1SIR). (**B**) Schematic representation of the mitochondrial degradation pathway of L-lysine, L-hydroxylysine, and L-tryptophan, highlighting the reaction catalyzed by GCDH and the accumulation of diagnostic metabolites in glutaric acidemia type 1 (GA1). Human GCDH (EC 1.3.8.6) functions as a homotetramer in the mitochondrial matrix and catalyzes the oxidative decarboxylation of glutaryl-CoA to crotonyl-CoA. During this reaction, electrons are transferred via the FAD cofactor to electron transfer flavoprotein (ETF), thereby reducing ETF from its oxidized (ETF_ox_) to its reduced (ETF_red_) form. Loss of GCDH activity leads to the accumulation of glutaryl-CoA and its hydrolysis products glutaric acid, 3-hydroxyglutaric acid, and glutaconic acid, as well as secondary formation of the acylcarnitines glutarylcarnitine (C5DC) and 3-hydroxydecanoylcarnitine (C10-OH).

Figure 1 summarizes the physiological role of human GCDH as a mitochondrial homotetramer catalyzing the oxidative decarboxylation of glutaryl-CoA with electron transfer flavoprotein (ETF). The figure also illustrates how the loss of GCDH activity results in the accumulation of glutaryl-CoA and its hydrolysis products (glutaric acid, 3-hydroxyglutaric acid, glutaconic acid), as well as disease-characteristic acylcarnitines such as glutarylcarnitine (C5DC) and 3-hydroxydecanoylcarnitine (C10-OH).

To date, more than 250 pathogenic variants have been identified in the GCDH gene located on chromosome 19p13.2, reflecting substantial genetic heterogeneity [4,5]. In untreated individuals, up to 90% of patients experience an acute encephalopathic crisis between 3 and 36 months of age, typically triggered by catabolic stress such as infection, vaccination, or surgical procedures [6]. These crises are characterized by bilateral striatal injury and often result in severe, persistent movement disorders, particularly dystonia and axial hypotonia, contributing to significant morbidity and mortality. A subset of patients develop neurological impairment without a documented acute crisis and are categorized as having an insidious disease course [7,8].

Macrocephaly is a frequent early clinical sign, but it may be overlooked or attributed to benign causes, which can delay diagnosis [9]. Because pre-crisis manifestations are often nonspecific, early identification based solely on clinical features is challenging [10,11]. For this reason, newborn screening programs using tandem mass spectrometry have been implemented in many countries to enable pre-symptomatic detection and timely initiation of therapy [12]. Standard management includes dietary lysine restriction, carnitine supplementation, and intensified metabolic support during intercurrent illness [13]. Early diagnosis and strict adherence to treatment have substantially reduced the incidence of encephalopathic crises; however, neurodevelopmental difficulties may still occur in some patients despite early intervention.

Although GA1 is primarily recognized as a neurometabolic disorder, transient transaminase elevations and hepatomegaly have been described anecdotally. However, systematic characterization of hepatic involvement is lacking. In Iran and neighboring countries with high rates of consanguinity, GA1 may present to hepatology services with unexplained hepatomegaly or abnormal liver function tests before the neurological phenotype fully evolves. Yet, published data regarding liver pathology in this context are scarce. To our knowledge, only a few reports from Iran have described the molecular spectrum of GCDH variants, providing limited information on hepatic manifestations. The aim of this study was to characterize the molecular spectrum of GCDH variants in a cohort of patients with GA1 who exhibited an unusual phenotype, including mild hepatopathy, and to explore potential genotype–phenotype correlations within this group.

## 2. Materials and Methods

### 2.1. Study Population, Data Collection, and Ethical Approval

This retrospective cohort study included all patients with suspected GA1 who underwent biochemical and molecular evaluation at Namazi Hospital (Shiraz, Iran) between December 2023 and December 2025 and were subsequently confirmed to have biallelic pathogenic or likely pathogenic GCDH variants. Data collection for this study was closed at the end of December 2025, and no additional patients were enrolled thereafter. Clinical information, biochemical results, histopathological findings, and diagnostic imaging data were collected retrospectively from electronic and paper medical records. Patients were included if they exhibited clinical features such as speech delay, neurological deficits, impaired growth and development during childhood, along with supportive biochemical findings, including elevated urinary GA and 3-OH-GA, and increased plasma C5DC levels. Patients were excluded only if key biochemical or genetic data were missing, which was the case for 3 initially identified individuals. All included patients were born to consanguineous parents, reflecting the high regional rate of consanguineous marriage; however, consanguinity was not used as a formal inclusion criterion. For the purpose of this study, mild hepatopathy was defined as either (i) persistent elevation of alanine aminotransferase (ALT) and/or aspartate aminotransferase (AST) above age-adjusted upper reference limits on at least two separate measurements, in the absence of hyperbilirubinemia, prolonged international normalized ratio, or clinical signs of liver failure, and/or (ii) histological evidence of portal or periportal fibrosis without cirrhosis or steatohepatitis on liver biopsy. Alternative causes of transaminase elevation (such as acute viral infection, drug-induced liver injury, and overt protein-energy malnutrition) were assessed through clinical history, medication review, anthropometric measurements, and, when clinically indicated, viral hepatitis serology and additional laboratory testing.

Neurodevelopmental status was extracted from routine clinical notes, including documented motor milestones, language development, and functional abilities; standardized scales (e.g., Bayley Scales of Infant Development or GMFCS) were not systematically applied.

Two independent members of the research team reviewed all medical records to extract data on clinical presentations, biochemical investigations, histopathology, and imaging. Written informed consent was obtained from the parents or legal guardians of all patients before their inclusion in the study. The study was approved by the Ethics Committee of Shiraz University of Medical Sciences (Approval No. IR.SUMS.REC.1404.030) and was conducted following the principles of the Declaration of Helsinki.

### 2.2. Biochemical Analyses

Free carnitine, plasma amino acid profile, and plasma acylcarnitine profile (including C5DC + C10-OH) were measured in EDTA-plasma using an AB Sciex 2200 liquid chromatography-tandem mass spectrometry (LC-MS/MS) (AB Sciex LLC, Framingham, MA, USA). Samples were prepared according to standard protocols with stable isotope-labeled internal standards and analyzed on a validated tandem mass spectrometry platform with routine calibration and internal quality controls. Urinary glutaric acid (GA) and 3-OH-GA were also quantified by LC-MS/MS following appropriate sample preparation. Reference ranges for key metabolites were as follows: urinary glutaric acid < 7 mmol/mol creatinine and urinary 3-OH-GA < 9 mmol/mol creatinine. The primary laboratory findings reported in Table 1 were obtained during the initial diagnostic workup, before initiation of the lysine-restricted diet and carnitine supplementation in all patients.

### 2.3. Whole-Exome Sequencing, Variant Annotation, and Interpretation

Whole-exome sequencing (WES) was conducted for all participants, and the resulting data were compared to the reference sequence of the *GCDH* gene (GenBank accession no. NM_000159.4). Exonic regions were enriched using the SureSelect Target Enrichment Reagent kit, PTN (#G9605A, Agilent Technologies, Santa Clara, CA, USA) and sequenced on an Illumina HiSeq 4000 platform, producing 101 bp paired-end reads with an average coverage depth of approximately 100× across the targeted regions. The raw sequencing data underwent standard processing, which included base calling, demultiplexing, alignment to the human reference genome (hg19), and variant calling. Reads were aligned to the human reference genome (hg19) using the Burrows-Wheeler Aligner with Maximal Exact Matches algorithm (BWA-MEM) v. 0.7.15. Variant calling was carried out with GATK HaplotypeCaller, followed by variant quality score recalibration and filtering in accordance with best practice guidelines. Variants were retained for further analysis if they had a depth of ≥10×, genotype quality of ≥20, and passed all standard quality filters.

Identified variants were analyzed for functional prediction using in silico tools such as Polymorphism Phenotyping v2 (PolyPhen-2), Sorting Intolerant From Tolerant (SIFT), and MutationTaster. The evolutionary conservation of the detected variants was assessed using Genomic Evolutionary Rate Profiling (GERP) and PhastCons scores. Population allele frequencies were determined using data from the Genome Aggregation Database (gnomAD) and the Iranome database [14]. Variant classification and interpretation were conducted following the guidelines of the American College of Medical Genetics and Genomics (ACMG) [15]. Variant pathogenicity was further evaluated using ensemble prediction scores (CADD and REVEL), where available. All sequence variants were reported according to the Human Genome Variation Society (HGVS) nomenclature [16]. In addition to GCDH, we also examined WES data for rare, protein-altering variants in a curated panel of genes associated with organic acidemias and primary mitochondrial disorders. No pathogenic or likely pathogenic variants were identified in these genes. All GCDH variants reported in this study were confirmed through bidirectional Sanger sequencing in the probands, and parental segregation analysis was performed when DNA samples were available.

### 2.4. In Silico Structural Modeling and Variant Mapping of GCDH

To map the location of individual variant amino acids in the human GCDH protein, the 3D crystal structure of human GCDH resolved by Fu and colleagues [17] was taken from the RCSB Protein Data Bank (PDB, access. no. 1SIR) and visualized using Ribbons XP version 3.0. To locate the structural context in which the variant amino acids are located in the GCDH protein, we performed in silico structure predictions with AlphaFold [18], using the human wild-type GCDH protein as a template.

## 3. Results

### 3.1. Clinical Manifestation

In this cohort, fifteen unrelated patients with infantile-onset GA1 were genetically confirmed. The group consisted of nine males and six females. The median age at diagnosis was 6 months (range: 6–8), and all patients initiated a lysine-restricted diet plus carnitine supplementation after diagnosis. The most common initial manifestations were macrocephaly, seizures, dystonia, and feeding difficulties. Motor impairment, developmental regression, hypotonia, and mild hepatopathy were also observed to varying degrees. One patient experienced an acute encephalopathic crisis with rapid neurological deterioration (Table 2). Liver biopsy was performed at a median age of 8 months (range 6–10) as part of the evaluation for hepatomegaly and/or elevated transaminases. Liver histopathology was available for all patients and revealed mild portal or periportal fibrosis (METAVIR F1–F2) in 10 out of 15 individuals (P5–P9 and P11–P15), whereas the remaining patients showed no relevant histological abnormalities (F0). Biochemically, liver transaminases were normal to mildly elevated, without evidence of hepatic synthetic dysfunction, consistent with a mild hepatopathy phenotype. Diagnostic brain MRI was not available for any patient. Therefore, structural hallmarks of GA1 such as striatal necrosis or frontotemporal atrophy could not be systematically assessed. The diagnosis of an acute encephalopathic crisis in P10 was based on the acute onset of severe hypotonia, loss of previously acquired motor skills, and feeding difficulties in the context of intercurrent illness, together with the characteristic biochemical profile, rather than on imaging confirmation of striatal lesions.

All patients were diagnosed after the age of 6 months and therefore started treatment later than recommended. After diagnosis, they were given standard management for GA1, which included a diet low in lysine, carnitine supplements, and emergency protocols for illnesses. However, treatment was only initiated after the delayed diagnosis.

### 3.2. Molecular Genetics Findings

Based on ACMG/AMP criteria, 14 patients carried variants classified as pathogenic and one carried a variant classified as likely pathogenic. All variants were present in the homozygous state; no compound heterozygous genotypes were observed. Molecular analysis revealed six distinct GCDH variants, all in the homozygous state. Missense variants were the most common (5/6), with a single nonsense variant identified. A common missense change, c.541G>C (p.Glu181Gln) in exon 6, was found in 11 out of 15 patients (73.3%), making it the most prevalent genotype in this series. Patients with this variant showed a similar clinical profile characterized by early macrocephaly and a movement disorder phenotype with seizures, dystonia, and mild hepatopathy. The other variants, c.1093G>A (p.Glu365Lys), c.679C>T (p.Arg227Trp), c.532G>A (p.Gly178Arg), and c.383G>A (p.Arg128Gln), each occurred in a single patient and were associated with specific clinical characteristics.

One patient carried a truncating variant, c.382C>T (p.Arg128Ter), and experienced an early acute encephalopathic crisis and significant developmental regression. All identified variants were located in coding exons, with exon 6 being a common mutation site in this group. These variants had been previously documented in ClinVar and/or HGMD, and were either absent or very rare in population databases, such as gnomAD (Table 2) [19,20,21].

### 3.3. Structure and Functional Implications of GCDH: In Silico Analysis

**Figure 2:**
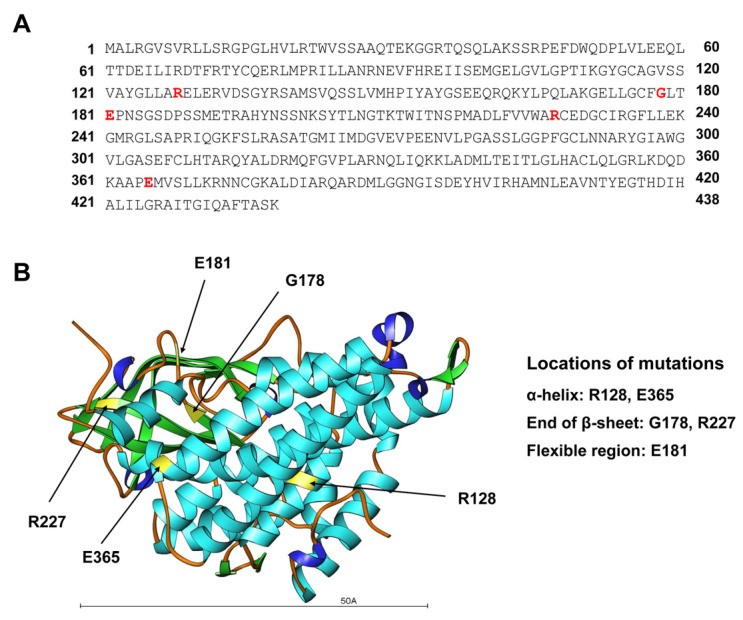
Mapping of GA1-associated missense variants onto the GCDH amino acid sequence and secondary-structure elements. (**A**) The primary amino acid sequence of human GCDH (residues 1–438) is displayed, with the five affected residues in this cohort (Arg128, Gly178, Glu181, Arg227, and Glu365) highlighted in red. The position of each residue along the polypeptide chain is indicated by the residue numbers at the beginning and end of each line. (**B**) A ribbon representation of the three-dimensional structure of a GCDH monomer, based on the crystal structure (PDB: 1SIR), is shown. In this representation, α-helices are depicted as cyan spirals, β-strands as green arrows, and flexible/non-regular regions (loops and turns) as orange coils. The positions of the five variant residues (R128, G178, E181, R227, and E365) are labeled to show that R128 and E365 are within α-helices, G178 and R227 are at the ends of β-sheets, and E181 is in a flexible loop region. The scale bar (50 Å ≈ 5 nm) provides an approximate measure of the size of the GCDH monomer. In simple terms, this figure shows where the altered amino acids are located along the protein chain and whether they lie in rigid (helices and sheets) or flexible regions of the enzyme.

Arg128 and Glu365 are located within α-helical segments, Gly178 and Arg227 are located at the ends of β-sheets, and Glu181 is positioned in a flexible region. This demonstrates that in our patients the recurring missense substitutions that lead to a loss of GCDH activity are located in distinct regions of the enzyme (Figure 2).

**Figure 3:**
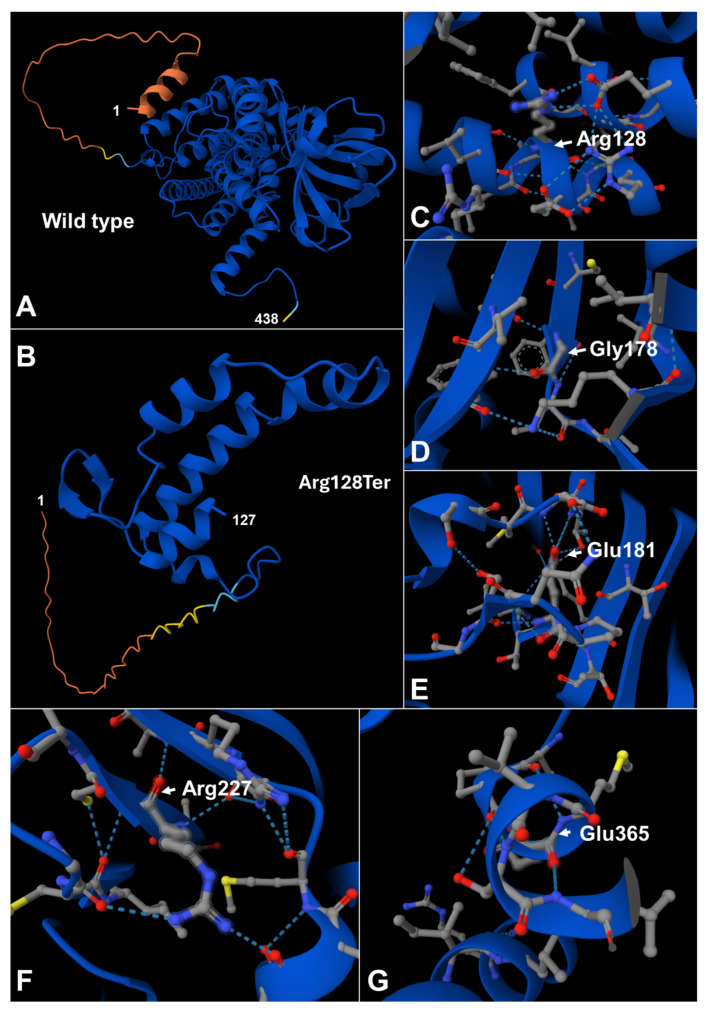
Structural consequences of GCDH variants predicted by in silico modeling. (**A**) A cartoon representation of the wild-type GCDH monomer, displaying the complete polypeptide chain from residue 1 at the N-terminus to residue 438 at the C-terminus. The compact catalytic core is in blue, while the extended N-terminal segment is colored from yellow to red to show its higher predicted flexibility. (**B**) The predicted structure of the truncated c.382C>T (p.Arg128Ter) variant, which includes only the N-terminal 127 residues and lacks the entire C-terminal part of the protein shown in panel A, demonstrating the significant structural loss expected for this nonsense mutation. (**C**–**G**) Close-up views of the local environments of the five missense residues Arg128, Gly178, Glu181, Arg227, and Glu365 in the context of the wild-type protein. The protein backbone is depicted as a blue ribbon, with side chains of the highlighted residues and neighboring amino acids shown as grey sticks with standard atom coloring (carbon in grey, oxygen in red, nitrogen in blue, sulfur in yellow), and hydrogen bonds or salt bridges indicated by light-blue dashed lines. These panels demonstrate that each residue is involved in a specific network of contacts that stabilize nearby α-helices, β-sheets, or loop regions, and that substitution at these positions is predicted to disrupt local packing and electrostatic interactions, ultimately decreasing overall GCDH stability and enzymatic activity.

To illustrate the expected effect of the truncating allele, we compared the full-length GCDH polypeptide with the significantly shortened product anticipated for the p.Arg128Ter variant. This nonsense variant is predicted to produce a truncated protein comprising only the N-terminal 127 amino acids, lacking the entire C-terminal portion of the enzyme (Figure 3). In Figure 3A, a linear schematic of the wild-type three-dimensional fold of the GCDH protein is depicted, while Figure 3B illustrates the proposed structure of the severely truncated c.382C>T (p.Arg128Ter) protein, highlighting the extent of structural loss linked to this nonsense variant. Figure 3C–G show the proposed structures of the protein regions surrounding each of the variant sites (Arg128, Gly178, Glu181, Arg227, and Glu365), emphasizing the specific local structural environments in which these amino acids are located. Upon visual inspection of these models, it appears that substitutions at these positions are likely to disrupt local side-chain packing, as well as attractive and repulsive interactions between adjacent residues, thereby impacting regional folding and, ultimately, influencing overall protein stability and enzymatic activity.

### 3.4. Biochemical Findings

Primary biochemical data for the 15 patients is summarized in Table 1. Plasma free carnitine concentrations were reduced or in the low-normal range in most patients (5.6–20.1 µmol/L), supporting secondary carnitine depletion as a common biochemical feature. Tandem mass spectrometry revealed increased C5DC levels across the cohort (0.45–1.76 µmol/mmol creatinine), while plasma amino acid profiles were unremarkable in all cases. Plasma amino acid profiles, including lysine, hydroxylysine, and tryptophan, were within age-adjusted reference ranges in all patients at the time of sampling. Urinary organic acid analysis demonstrated markedly elevated GA and 3-OH-GA excretion in all patients. Urinary GA levels ranged from 89 to 297 mmol/mol creatinine, and urinary 3-OH-GA ranged from 56 to 201 mmol/mol creatinine. The highest metabolite levels were observed in patients with more clinical involvement, including those with hepatopathy or severe neurological features. Notably, patients P1–P4, who did not show clinical or histological evidence of hepatopathy, had comparatively lower C5DC concentrations and urinary GA and 3-OH-GA excretion than patients P5–P15, suggesting a trend toward higher metabolite burden in individuals with more extensive neurological and hepatic involvement, although the small cohort size precludes formal statistical analysis.

Liver transaminases were within or mildly above age-adjusted reference ranges in most patients. Alanine aminotransferase (ALT) ranged from 8 to 49 U/L and aspartate aminotransferase (AST) from 7 to 40 U/L, with modest elevations observed in a subset of patients, particularly those carrying the common c.541G>C variant. Alkaline phosphatase (ALP) levels were variably elevated (190–634 U/L), consistent with the young age of the cohort. Serum albumin concentrations were largely preserved, although mild hypoalbuminemia was noted in several patients.

## 4. Discussion

In this cohort of 15 unrelated patients with infantile-onset GA1, we present a comprehensive clinical, molecular, and biochemical analysis that highlights a significant predominance of a single common *GCDH* variant, c.541G>C (p.Glu181Gln). The uniformity of both genotype and phenotype in this group provides valuable insights into regional mutational patterns, genotype–phenotype correlations, and potential founder effects in GA1. Importantly, all patients in this series were diagnosed and treated after 6 months of age, which is considerably later than the time window targeted by newborn screening and current management guidelines. Therefore, the relatively high frequency of motor impairment and developmental delay observed here likely reflects, at least in part, the consequences of delayed initiation of metabolic therapy, limiting our ability to attribute neurological severity solely to specific GCDH variants.

The most striking discovery is the frequent occurrence of the homozygous c.541G>C (p.Glu181Gln) variant, found in 11 of 15 patients (73.3%). All affected individuals were homozygous, indicating autosomal recessive inheritance and suggesting a possible founder effect or regional concentration. The clustering of this variant within exon 6, identified as a mutational hotspot in our cohort, further suggests a non-random distribution of disease-causing alleles in this population. While the c.541G>C (p.Glu181Gln) variant has been previously documented in international databases (ClinVar, HGMD) and observed in patients from Turkey and Germany, its prevalence in our study raises the likelihood that this variant may have originated or expanded within a specific ancestral group [20,22]. The high proportion of unrelated patients who are homozygous for p.Glu181Gln in a setting of frequent consanguinity is consistent with the presence of a common ancestral allele in this region. However, a founder effect remains speculative in the absence of haplotype data. Future studies should include haplotype analysis to definitively confirm this hypothesis.

Despite minor individual differences, patients carrying the common c.541G>C (p.Glu181Gln) variant exhibited a consistent clinical profile characterized by early-onset macrocephaly, dystonia, seizures, and varying levels of motor impairment. This clustering of symptoms highlights a notable genotype–phenotype correlation linked to this recurring allele in our cohort. Because neurodevelopmental status was derived from descriptive clinical documentation rather than standardized assessments, inferences regarding the relationship between specific variants and the severity of motor or cognitive impairment must be interpreted with caution. Within this cohort, patients carrying the p.Glu181Gln variant frequently exhibited mild biochemical and/or histological liver abnormalities, suggesting that subtle hepatic involvement may accompany the classical neurological phenotype in a subset of GA1 patients [20,21]. Liver issues in GA1 have generally been considered secondary or clinically insignificant compared to the severe neurological symptoms. These findings suggest that p.Glu181Gln may confer a unique metabolic vulnerability affecting hepatic mitochondrial function in addition to cerebral energy metabolism. The notion that p.Glu181Gln might confer a particular vulnerability of hepatic mitochondria is speculative and currently based only on the recurrent observation of mild hepatopathy in our small cohort; functional studies examining the impact of this variant on GCDH activity and mitochondrial homeostasis in hepatocyte models will be required to substantiate this hypothesis. Given the small sample size and lack of a control group, these observations should be interpreted cautiously and regarded as hypothesis-generating.

The histological demonstration of mild liver fibrosis in the majority of our patients is striking, especially considering that routine liver function tests were normal or only slightly elevated. This emphasizes that conventional biochemical markers may underestimate chronic hepatic involvement in GA1. The observation that fibrosis occurred across a range of ages and tended to co-occur with higher metabolite levels suggests that sustained metabolic dysregulation might contribute to subclinical liver injury. However, larger longitudinal studies will be required to determine which patients are at risk and whether this is related to specific genotypes, such as p.Glu181Gln.

In contrast, the single truncating variant (c.382C>T; p.Arg128Ter) was associated with an early acute encephalopathic crisis and marked developmental regression, consistent with a more severe loss-of-function effect as reported previously [20]. This observation aligns with the expected impact of nonsense variants leading to premature termination and likely nonsense-mediated decay. The comparison between the truncating mutation and the common missense variant further supports a mutation-dependent spectrum of clinical severity.

Our in silico structural analysis of GCDH, based on the available crystal structure and AlphaFold predictions, provides a mechanistic framework for the genotype–phenotype correlations observed in this cohort. Mapping the five missense residues onto defined secondary structure elements showed that Arg128 and Glu365 are embedded within α-helices, Gly178 and Arg227 are located at the termini of β-sheets, and Glu181 resides in a flexible region, indicating that functionally important interactions are distributed across distinct structural motifs of the enzyme. As illustrated in Figure 2 and Figure 3, visual inspection of the modeled variants suggests that each substitution perturbs local side-chain packing and electrostatic interactions, which is expected to compromise regional folding and, ultimately, overall protein stability and catalytic activity. In this context, the common p.Glu181Gln change within a flexible segment may primarily alter local dynamics rather than completely abolish the global fold, consistent with the relatively homogeneous but not fulminant infantile-onset phenotype with mild hepatopathy in our patients. By contrast, the p.Arg128Ter nonsense variant is predicted to yield a severely truncated polypeptide comprising only the N-terminal 127 amino acids, lacking the remainder of the C-terminal portion of the enzyme, and thus most of the structural elements required for proper folding and enzymatic function, providing a plausible structural explanation for the early encephalopathic crisis and marked developmental regression in this individual. Together, these models support the notion that missense variants in structurally constrained helices or β-sheet termini act as destabilizing hypomorphic alleles, whereas premature termination at Arg128 represents a near-null allele, mirroring the clinical spectrum from relatively uniform infantile-onset disease with mild hepatopathy to severe early neurodegeneration. More broadly, our findings illustrate how integration of three-dimensional protein modeling with clinical and biochemical data can refine the variant interpretation of the American College of Medical Genetics and Genomics (ACMG), particularly in populations where predominant alleles such as p.Glu181Gln predominate.

It is important to note that the identification of a predominant pathogenic allele in infantile-onset GA1 has significant implications for diagnosis, surveillance, and genetic counseling. In regions where the c.541G>C variant is enriched, targeted molecular screening could accelerate confirmation of GA1 in infants presenting with macrocephaly and movement disorders, enabling earlier initiation of lysine-restricted diets, carnitine supplementation, and emergency protocols that substantially reduce the risk of irreversible striatal injury. Additionally, the consistent observation of mild but common hepatic involvement in individuals carrying the p.Glu181Gln variant indicates that clinical monitoring should extend beyond the neurological domain to include periodic assessment of liver enzymes and function, even in the absence of overt hepatic disease. By delineating a population-specific mutational pattern and its associated clinical features, this study expands the global understanding of *GCDH* variant spectra and highlights the value of regional genetic data for rare metabolic disorders. The clustering of c.541G>C in our cohort supports the development of regional mutation databases and international registries to clarify founder effects, migration patterns, and genotype–phenotype variability [23]. Future work incorporating haplotype analysis, functional assays of residual enzyme activity, and longitudinal outcome studies will be essential to confirm a founder origin, refine risk stratification, and optimize personalized management and newborn screening strategies in high-prevalence settings.

Moreover, the biochemical profile across the cohort was highly consistent with classical GA1. All patients demonstrated markedly elevated urinary GA and 3-OH-GA, alongside increased C5DC levels on tandem mass spectrometry. Plasma amino acid profiles were unremarkable, and free carnitine levels were reduced or low–normal in most cases, supporting secondary carnitine depletion as a common metabolic consequence. Taken together, these findings confirm a consistent metabolic signature across the cohort and strongly support the diagnosis of GA1, with modest variability in metabolite burden and liver-related parameters. Notably, higher urinary GA and 3-OH-GA levels tended to cluster in patients with more pronounced neurological impairment or hepatopathy, suggesting that metabolite load may partially correlate with clinical severity. However, the relatively narrow biochemical range observed in patients with the common p.Glu181Gln variant reinforces the impression of a biochemically and clinically homogeneous subgroup. It should be noted that the reduced albumin concentrations observed in some patients may be multifactorial and reflect not only mild hepatic dysfunction but also poor nutritional intake due to chronic feeding difficulties and intercurrent illness; importantly, there was no evidence of nephrotic-range proteinuria in the available data.

## 5. Limitations of the Study

This study has several limitations that should be considered when interpreting the findings. First, the cohort is relatively small (n = 15) and recruited from a single tertiary center in Shiraz, Iran. This may limit generalizability and mainly reflect local referral patterns and high consanguinity rather than the broader GA1 population. Second, data were collected retrospectively over a limited observation period, so clinical histories, especially long-term neurological and hepatic outcomes may be incomplete or biased. Third, neurological status and development were inferred from routine clinical documentation without standardized rating scales, and brain MRI was not systematically available, which restricts detailed characterization of the neurological phenotype and correlation of genotypes with structural brain changes. Fourth, we did not perform functional assays (e.g., enzyme activity measurements or in vitro expression studies) or haplotype analysis. Therefore, the predicted pathogenicity of variants, particularly p.Glu181Gln, and the suggested founder effect remain inferential and rely on in silico modeling and population frequency data. Fifth, we did not include a comparison group of GA1 patients without hepatopathy or healthy controls. The small sample size allowed only descriptive analyses without formal statistical testing. Therefore, observed associations between genotype, metabolite burden, and clinical severity should be regarded as hypothesis-generating rather than definitive.

## 6. Conclusions

In conclusion, our findings reveal a striking predominance of the homozygous c.541G>C (p.Glu181Gln) GCDH variant among patients with infantile-onset GA1 in this cohort, suggesting a potential founder effect or regional enrichment. The cohort demonstrated a largely homogeneous early-onset neurological phenotype consistent with classical GA1, along with a consistent metabolic signature characterized by elevated disease-specific metabolites, relative carnitine deficiency, and preserved plasma amino acid patterns. The unexpected observation of mild but common hepatic involvement in individuals with the p.Glu181Gln variant expands the recognized phenotypic spectrum and warrants further investigation. Therefore, these data provide clinically relevant insights into genotype–phenotype correlations and emphasize the importance of population-tailored genetic and metabolic evaluation in GA1.

## Figures and Tables

**Table 1:** Primary laboratory findings in patients with glutaric acidemia type 1 (GA1) (n = 15) *.

Patient	Plasma Free Carnitine (µmol/L)	C5DC + C10-OH (µmol/mmol Cr)	Plasma Amino Acids (LC-MS/MS)	Urinary GA (mmol/mol Cr)	Urinary 3-OH-GA (mmol/mol Cr)	ALT (U/L)	AST (U/L)	ALP (U/L)	Albumin (g/dL)
P1	19.5	0.81	Normal	101	88	12	10	518	4.5
P2	12.5	0.95	Normal	92	71	8	7	612	4.1
P3	14.1	0.45	Normal	89	56	15	12	634	4.1
P4	20.1	0.65	Normal	170	102	19	15	423	4.0
P5	15.5	1.76	Normal	297	166	39	33	412	3.5
P6	8.5	1.45	Normal	254	190	39	35	389	3.1
P7	8.5	1.32	Normal	198	150	41	37	370	2.9
P8	7.9	1.70	Normal	213	150	41	35	430	3.1
P9	7.6	1.66	Normal	188	169	40	38	512	2.8
P10	5.6	0.98	Normal	105	102	21	12	190	4.0
P11	8.2	1.20	Normal	211	176	43	37	550	2.7
P12	11.0	1.43	Normal	215	201	46	40	545	3.4
P13	13.2	1.29	Normal	243	191	47	40	612	2.5
P14	8.5	0.98	Normal	201	167	49	39	632	3.5
P15	7.1	1.02	Normal	214	146	45	36	615	2.8

* All biochemical measurements were obtained during the initial diagnostic work up, before the initiation of a lysine-restricted diet and carnitine supplementation. Abbreviations and reference ranges: Plasma free carnitine: 7.6–32.0 µmol/L; glutarylcarnitine + 3-hydroxydecanoylcarnitine (C5DC + C10-OH): <0.15 µmol/mmol creatinine; glutaric acid (GA): <7 mmol/mol creatinine; 3-hydroxyglutaric acid (3-OH-GA): <9 mmol/mol creatinine; alanine aminotransferase (ALT) in children: 4–36 U/L; aspartate aminotransferase (AST): 8–33 U/L; alkaline phosphatase (ALP): <645 U/L; Albumin: 3.8–5.4 g/dL.

**Table 2:** Molecular and clinical characteristics of patients with infantile-onset glutaric acidemia type 1 (GA1) (n = 15).

Patient	Sex/Age at Diagnosis (M)	Age at Treatment Initiation (M)	Clinical Features	Nucleotide Change ^1^	Protein Change	Exon	Variant Type	Zygosity	ACMG Class ^2^	ClinVar ID	gnomAD AF ^3^	Prior Report ^4^
P1	M/6	7	Macrocephaly, feeding difficulty	c.1093G>A	p.(Glu365Lys)	11	Missense	Hom	Pathogenic	VCV000002086.29	Rare (<0.0001)	HGMD
P2	F/6	6.5	Feeding difficulty, dystonia	c.679C>T	p.(Arg227Trp)	8	Missense	Hom	Pathogenic	VCV000658536.10	Rare	Absent
P3	M/6	7	Feeding difficulty, dystonia	c.532G>A	p.(Gly178Arg)	6	Missense	Hom	Pathogenic	VCV000371271.21	Rare	HGMD
P4	M/7	9	Macrocephaly, ataxia, cognitive decline	c.383G>A	p.(Arg128Gln)	6	Missense	Hom	Likely pathogenic	VCV000189063.21	Rare	HGMD
P5	F/6	8	Macrocephaly, seizures, dystonia, irritability, mild hepatopathy	c.541G>C	p.(Glu181Gln)	6	Missense	Hom	Pathogenic	VCV000522644.6	0.00002	HGMD
P6	F/6	8	Macrocephaly, seizures, dystonia, irritability, mild hepatopathy	c.541G>C	p.(Glu181Gln)	6	Missense	Hom	Pathogenic	VCV000522644.6	0.00002	HGMD
P7	F/6	7	Macrocephaly, seizures, dystonia, mild hepatopathy	c.541G>C	p.(Glu181Gln)	6	Missense	Hom	Pathogenic	VCV000522644.6	0.00002	HGMD
P8	M/6	6.5	Macrocephaly, seizures, dystonia, mild hepatopathy	c.541G>C	p.(Glu181Gln)	6	Missense	Hom	Pathogenic	VCV000522644.6	0.00002	HGMD
P9	M/6	7	Macrocephaly, seizures, dystonia, mild hepatopathy	c.541G>C	p.(Glu181Gln)	6	Missense	Hom	Pathogenic	VCV000522644.6	0.00002	HGMD
P10	M/8	9	Acute encephalopathic crisis, hypotonia, developmental regression, feeding difficulty	c.382C>T	p.(Arg128Ter)	6	Nonsense	Hom	Pathogenic	VCV000371176.12	Absent	HGMD
P11	F/7	9	Macrocephaly, seizures, dystonia, motor impairment, mild hepatopathy	c.541G>C	p.(Glu181Gln)	6	Missense	Hom	Pathogenic	VCV000522644.6	0.00002	HGMD
P12	F/7	8	Macrocephaly, seizures, dystonia, motor impairment, mild hepatopathy	c.541G>C	p.(Glu181Gln)	6	Missense	Hom	Pathogenic	VCV000522644.6	0.00002	HGMD
P13	M/6	7	Macrocephaly, seizures, dystonia, motor impairment, mild hepatopathy	c.541G>C	p.(Glu181Gln)	6	Missense	Hom	Pathogenic	VCV000522644.6	0.00002	HGMD
P14	M/8	10	Macrocephaly, seizures, dystonia, motor impairment, mild hepatopathy	c.541G>C	p.(Glu181Gln)	6	Missense	Hom	Pathogenic	VCV000522644.6	0.00002	HGMD
P15	M/8	9	Macrocephaly, seizures, dystonia, motor impairment, mild hepatopathy	c.541G>C	p.(Glu181Gln)	6	Missense	Hom	Pathogenic	VCV000522644.6	0.00002	HGMD

^1^ Variants were reported according to HGVS using GCDH NM_000159.4; ^2^ ACMG classification was based on ACMG/AMP 2015 criteria; ^3^ AF = Allele frequency in gnomAD; ^4^ All variants were previously reported. Abbreviations used are: HGMD, Human Gene Mutation Database (available at: https://www.hgmd.cf.ac.uk/ac/index.php, last accessed 10 April 2026); Hom, homozygous; M, month(s); P = patient.

## Data Availability

The original contributions presented in this study are included in the article. Further inquiries can be directed to the corresponding authors.

## Abbreviations

The following abbreviations are used in this manuscript:

3-OH-GA	3-hydroxyglutaric acid
ACMG	American College of Medical Genetics and Genomics
ACMG/AMP	American College of Medical Genetics and Genomics/Association for Molecular Pathology
AF	Allele frequency
ALP	Alkaline phosphatase
ALT	Alanine aminotransferase
AST	Aspartate aminotransferase
C10-OH	3-hydroxydecanoylcarnitine
C5DC	Glutarylcarnitine
Cr	Creatinine
EC	Enzyme Commission
ETF	Electron transfer flavoprotein
ETFred	Reduced electron transfer flavoprotein
ETFox	Oxidized electron transfer flavoprotein
GA	Glutaric acid
GA1	Glutaric acidemia type 1
GCDH	Glutaryl-CoA dehydrogenase
GERP	Genomic Evolutionary Rate Profiling
gnomAD	Genome Aggregation Database
HGMD	Human Gene Mutation Database
HGVS	Human Genome Variation Society
Hom	Homozygous
LC-MS/MS	Liquid chromatography–tandem mass spectrometry
MRI	Magnetic resonance imaging
OMIM	Online Mendelian Inheritance in Man
P	Patient
PDB	Protein Data Bank
SIFT	Sorting Intolerant From Tolerant
WES	Whole-exome sequencing
